# Real-world 6-month outcomes of minimally invasive aortic valve replacement with the EDWARDS INTUITY Elite valve system

**DOI:** 10.1093/icvts/ivac083

**Published:** 2022-04-08

**Authors:** Günther Laufer, Justus T Strauch, Kim A Terp, Marco Salinas, Jose M Arribas, Massimo Massetti, Martin Andreas, Christopher P Young

**Affiliations:** 1 Department of Cardiac Surgery, Medical University of Vienna/Cardiac Surgery, Vienna, Austria; 2 Department of Cardiothoracic Surgery, University Hospital Bergmannsheil Bochum, Bochum, Germany; 3 Department of Cardiac Surgery, Aarhus University Hospital Skejby, Aarhus, Denmark; 4 Department of Cardiac Surgery, Ospedale Del Cuore G.Pasquinucci, Massa, Italy; 5 Department of Cardiac Surgery, Hospital Clínico Universitario Virgen de la Arrixaca, El Palmar, Murcia, Spain; 6 Department of Cardiovascular Sciences, Catholic University of Sacred Heart—Rome, Rome, Italy; 7 Department of Cardiac Surgery, Medical University of Vienna, Vienna, Austria; 8 Department of Cardiothoracic Surgery, St Thomas' Hospital, London, UK

**Keywords:** Aortic valve replacement, Rapid-deployment valve, Sutureless valve, Bioprosthesis, Heart valve, Haemodynamics

## Abstract

**OBJECTIVES:**

We report on real-world safety and performance outcomes of minimally invasive rapid-deployment aortic valve replacement using the EDWARDS INTUITY Elite aortic valve system.

**METHODS:**

The study valve system was used in a European, prospective, multicentre post-market study. Various procedural, haemodynamic and clinical outcomes were evaluated through 6 months of post-implant.

**RESULTS:**

A total of 276 patients out of 280 (98.6%) enrolments were successfully implanted with the study valve using a minimally invasive approach between February 2016 and April 2017. Of these 276 patients, 240 (87%) underwent partial sternotomy and 36 (13%) patients underwent right thoracotomy. Mean cross-clamp time was 51.9 [standard deviation (SD): 16.0] min. From baseline to 6 months, the mean effective orifice area increased from 0.8 (SD: 0.3) to 1.8 (SD: 0.6) cm^2^ and the mean systolic gradient decreased from 46.0 (SD: 14.1) to 8.8 (SD: 3.7) mmHg. After 6 months, 70.7% and 26.4% of patients were in New York Heart Association class I and II, respectively. Freedom from death, major bleeding, major paravalvular leak, reoperation and device explant at 6 months were 96.0%, 98.5%, 98.8%, 99.2% and 99.2%, respectively.

**CONCLUSIONS:**

These results demonstrate that the study valve is a safe and effective choice for patients undergoing aortic valve replacement via minimally invasive surgery.

**Name and registration of registry:**

MISSION (Assessing clinical outcomes using the EDWARDS INTUITY Elite Valve System in isolated AVR using Minimally InvaSive Surgery In a EurOpean multi-ceNter, active, post-market registry). clinicaltrials.gov ID #NCT02907463.

**Figure:**
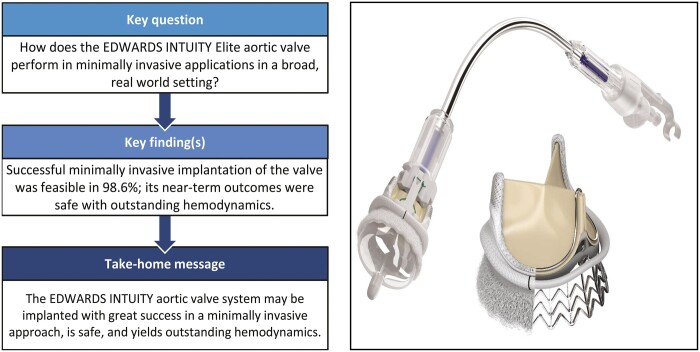

## INTRODUCTION

Several treatment options are currently evaluated for the treatment of aortic valve stenosis, which is the most common heart valve disease requiring intervention. While conventional surgical valve replacement via full sternotomy was seen as the golden standard 20 years ago, several technological developments significantly changed the armamentarium for the treatment of aortic stenosis nowadays.

The EDWARDS INTUITY valve system (Edwards Lifesciences, Irvine, CA, USA) is one of the 2 currently available sutureless and rapid-deployment aortic valves. It represents the combination of the long-lasting Edwards Magna bioprosthesis with a stent-based balloon-expandable fixation system [1, 2]. This enables faster implantation after surgical debridement and supports minimally invasive implantation techniques [3, 4].

This valve was initially evaluated in the TRITON study and later in the FOUNDATION registry, which both revealed favourable short- and intermediate-term results [1, 2]. Furthermore, excellent intermediate-term survival was shown in a single-centre observational registry (Vienna Intuity Comprehensive Evaluation—VICE registry) [2, 5]. Comparative retrospective studies indicated that this valve technology is increasingly applied in minimally invasive procedures [4]. Furthermore, one randomized clinical trial (CADENCE-MIS) showed superior results regarding operative times via minimally invasive surgery compared to conventional bioprosthesis via full sternotomy [3]. Therefore, Edwards Lifesciences initiated the MISSION (Assessing clinical outcomes using the EDWARDS INTUITY Elite Valve System in isolated aortic valve replacement (AVR) using Minimally InvaSive Surgery In a EurOpean multi-ceNter, active, post-market registry) trial to specifically evaluate the outcome of the INTUITY Elite valve system in patients operated exclusively with a minimally invasive access. Presented here are the final study results.

## PATIENTS AND METHODS

### Ethics statement

Formal, written consent was obtained from all trial participants prior to participation in any clinical trial activities. The first investigational centre achieving Ethics Committee approval was Murcia Health Department, ‘Virgen de la Arrixaca’ Hospital Clinical Research Ethics Committee, Murcia, Spain, Approval #2015-11-4-HCUVA, approved on 21 December 2015.

### Study population

The MISSION registry was a prospective, single-arm, multicentre, post-market, 6-month follow-up study of the EDWARDS INTUITY Elite rapid-deployment valve system in Europe. The study enrolled patients who were 18 years or older and were candidates for AVR due to aortic stenosis or mixed aortic stenosis and aortic insufficiency disease. The decision to implant the study valve in each patient was made independently and in advance of the data collection of this study. Patients were selected for minimally invasive AVR according to local protocols; all units were experienced in minimally invasive AVR. The study protocol was approved by each investigational centre’s local ethics committee and written informed consent was provided by all study subjects. The study was registered as clinicaltrials.gov identifier #NCT02907463. The study was funded by Edwards Lifesciences.

Those patients with a history of active endocarditis or myocarditis within 3 months of the scheduled operation or those diagnosed with pure aortic insufficiency or aneurysm of the aortic root or ascending aorta were excluded.

### Implant procedure and follow-up

Patients were implanted with the valve system under a minimally invasive approach at the discretion of the surgeon; ultimately, the minimally invasive approaches were either partial sternotomy (PS) or right anterior thoracotomy (RAT). The valve system included the EDWARDS INTUITY Elite Aortic Valve (Model 8300AB), available in sizes between 19 and 27 mm, and the EDWARDS INTUITY Elite Delivery System (Model 8300DB). An oblique hockey-stick aortotomy crossing the sinotubular junction was carried out with an extension to the middle of the non-coronary sinus. Excision of the native leaflets and debridement of the annulus and LVOT was then completed. After placing the sizer barrel into the annulus, the choice of the proper valve size was made through testing of the next smaller and larger valve sizers. Three non-pledgeted guiding sutures were equidistantly placed within the native annulus, ensuring that placement of the sutures corresponded to the markers on the sewing cuff. The valve system was then parachuted and securely seated on the annulus. The balloon catheter was advanced within the delivery system until it snapped into place. The balloon was inflated to 4.5–5.0 atmospheres, per the device instructions for use, and the stent frame was deployed in a rapid fashion. The prosthesis was thereafter placed in a supra-annular position with the stent frame below the annulus in a flared configuration. The 3 sutures were tied, then cut close to the knots, and the delivery system and valve holder were removed as a single unit. The aortotomy was closed in the usual manner. Postoperative anticoagulation was left to the investigator’s discretion.

Patient follow-up included telephone assessment at 30 days and a clinic visit at 6 months post-implant. Safety outcomes were evaluated at 30 days and 6 months. Valve haemodynamic end-points were evaluated at baseline, discharge and 6 months. New York Heart Association (NYHA) functional class assessments were collected at baseline, 1 and 6 months. Finally, quality of life was measured using the SF-36 and EQ-D5 instruments at baseline and at 6 months.

### Study end-points

At the time this registry was originally designed, its primary end-point and hypothesis were that the study valve would exhibit a reduced aortic cross-clamp time compared to conventional, sutured stented valves in a minimally invasive setting, as per such published reports. Upon the registry’s conclusion, however, this primary end-point was felt to be too limiting; therefore, a comprehensive descriptive perspective of the study valve’s performance is here reviewed.

Safety end-points were selected and defined according to the standardized classifications of the Society of Thoracic Surgeons, the American Association for Thoracic Surgery and the European Association of Cardio-Thoracic Surgery, per Akins *et al.* [6]. They included all-cause mortality, study valve-related mortality, thromboembolism, haemolysis, endocarditis, study valve thrombosis, major paravalvular leak (PVL), bleeding, study valve explant or reoperation, deterioration of study valve (structural and non-structural), renal and respiratory failure, deep sternal wound infection and pacemaker implantation. Events within 30 days of the index surgery were classified as early events; those occurring after 30 days were reported as late events. Major PVL was any PVL, exclusive of that associated with thrombosis or infection, which led to intervention, reoperation or was considered a serious adverse event. All safety events were reviewed and adjudicated by an independent Clinical Events Committee.

Valve haemodynamic end-points included mean and peak pressure gradient, and effective orifice area (EOA). Severe patient–prosthesis mismatch (PPM), defined as an EOA index <0.65 cm^2^/m^2^, was assessed at 6 months. Only haemodynamic data evaluated by an independent echocardiography core laboratory were analysed. Other effectiveness end-points included aortic cross-clamp time (XCT), cardiopulmonary bypass time (CPBT), haemodynamic performance and NYHA functional class at 6 months. Device technical success was defined as leaving the operating room with the successful deployment of the study valve via minimally invasive surgery and retrieval of the delivery system. Procedural technical success was reported as a successful implant with no adverse events necessitating a reoperation, implant of permanent pacemaker (with baseline sinus rhythm and no other conduction issues), or subject valve-related death within 10 days of the index procedure or discharge, whichever occurred first.

### Data management and statistical analysis

The investigational sites collected and recorded the clinical data. Edwards Lifesciences, the study sponsor, monitored and aggregated the clinical data, and analysed them per the protocol and statistical analysis plan. The investigators were responsible for an accurate accounting of these data as represented in this report. Summary statistics for categorical variables include the number and percentage of subjects with a recorded value for the variable of interest. Continuous variables are reported as mean (standard deviation). Early safety event rates are reported as the percentage of patients with the early event among those patients successfully undergoing implantation of the study valve under minimally invasive surgery. Late safety event rates are also expressed as a percentage per late patient-year of follow-up. Kaplan–Meier analysis was carried out to evaluate the freedom from event rate of each safety outcome. Preoperative values of aortic EOA, left ventricular ejection fraction, mean systolic gradient and peak systolic gradient were compared to their respective values at 6 months via paired *t*-tests and adjusted for multiple comparisons using a Bonferroni correction. NYHA classification distribution at baseline was compared to that at 6 months using the marginal homogeneity test after converting NYHA Class to numeric values (class I = 1, class II = 2, class III = 3 and class IV = 4). The proportion of patients whose NYHA class improved at 1 and 6 months compared to baseline were evaluated using a binomial test. Components of the SF-36 Quality of Life instrument were evaluated at 6 months compared to baseline using paired Student’s *t*-tests; normality of the continuous variables was evaluated via graphical assessments including histograms and Q–Q plots. For all statistical analyses, a *P*-value of <0.05 was considered statistically significant. No imputation was carried out for missing values. SAS version 7 (SAS Institute Inc, Cary, NC, USA) was used for all statistical analyses.

## RESULTS

### Baseline characteristics

The registry enrolled 280 patients at 22 centres in Europe between February 2016 and April 2017, and the final patient follow-up was November 2017. The patient cohort was generally consistent with that of a typical AVR population. For the enrolled cohort, the mean age was 73.7 [standard deviation (SD): 6.8) years, 51.3% were female and the EuroSCORE II was 1.8% (SD: 1.3]. Baseline characteristics for the per-protocol cohort are summarized in Table 1.

**Table 1:** Preoperative characteristics of the patient cohort in whom the study valve was successfully implanted via a minimally invasive procedure

Characteristic	Summary
Age (years) *n*: mean ± SD (min to max)	
Age	276: 73.7 (SD: 6.8) (44.0–96.0)
Age distribution	
18–39 years	0.0% (0/276)
40–49 years	0.4% (1/276)
50–59 years	3.3% (9/276)
60–69 years	21.0% (58/276)
60–64 years	4.3% (12/276)
65–69 years	16.7% (46/276)
70–79 years	58.3% (161/276)
≥80 years	17.0% (47/276)
Sex % (*n*/*N*)	
Female	51.4% (142/276)
Male	48.6% (134/276)
BMI	275: 29.3 (SD: 5.0) (16.1–47.3)
BMI Category % (*n*/*N*)	
<18.5 kg/m^2^	0.7% (2/275)
18.5–24.9 kg/m^2^	17.1% (47/275)
25.0–30.0 kg/m^2^	39.3% (108/275)
>30 kg/m^2^	42.9% (118/275)
NYHA class I	4.0% (11/275)
NYHA class II	44.7% (123/275)
NYHA class III	49.1% (135/275)
NYHA class IV	2.2% (6/275)
EuroSCORE II (%)	259: 1.8 (SD: 1.3) (0.1–13.2)

BMI: body mass index; EuroSCORE: European System for Cardiac Operative Risk Evaluation; NYHA: New York Heart Association.

### Procedural results

**Figure 1:**
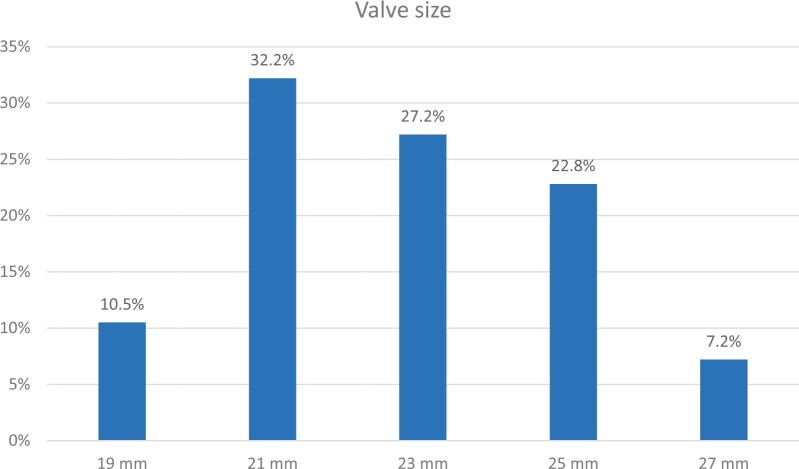
Valve size distribution. The bars show the proportion of patients implanted with each valve size.

The study valve was successfully implanted via minimally invasive surgery in 276 (98.6%) of the 280 enrolled patients (2 subjects received the study valve through a conventional full sternotomy and 2 others received a non-study commercial valve). Unless otherwise noted, the remaining results reported here are based upon the primary study cohort of 276 subjects successfully implanted with the study valve via minimally invasive surgery. Study valve sizes are shown in Fig. 1 and were 19 mm in 10.5% (29/276), 21 mm in 32.2% (89/276), 23 mm in 27.2% (75/276), 25 mm in 22.8% (63/276) and 27 mm in 7.2% (20/276). The mean prosthesis size was 22.5 (SD: 2.2) mm (median: 23 mm).

**Figure 2:**
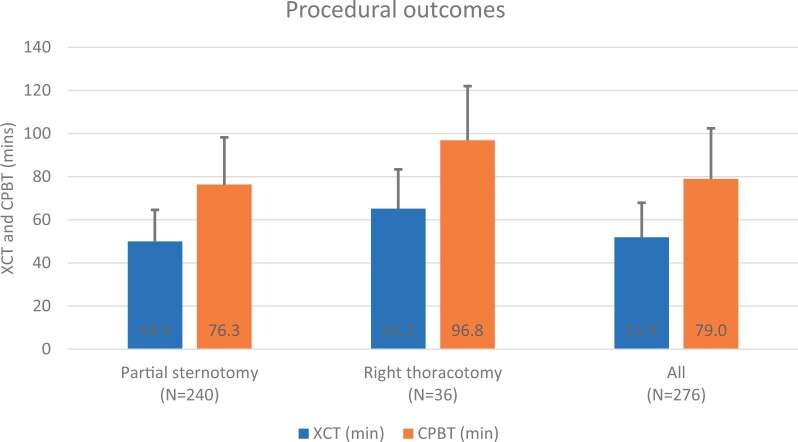
Procedural outcomes. The bars show the mean ± standard deviation XCT and CPBT minutes. CPBT: cardiopulmonary bypass time; XCT: aortic cross-clamp time.

Two hundred and forty (87%) of the 276 patients underwent PS and 36 (13%) patients underwent RAT (Fig. 2). Device technical success among the enrolled cohort of 280 patients was 98.6% [276/280 (first attempt success in 270 patients; second attempt success in 6 patients)], and procedural success was 97.9% (274/280).

The XCT for the study cohort was 51.9 (SD: 16.0) min (Fig. 2). The XCT for patients undergoing PS was 49.9 (SD: 14.7) min, compared to 65.2 (SD: 18.2) min for those undergoing RAT. The cardiopulmonary bypass time averaged 79.0 (SD: 23.4) min; it was 76.3 (SD: 21.9) min and 96.8 (SD: 25.2) min for the PS and RAT groups, respectively. Skin-to-skin procedure time was 172.8 (SD: 40.7) min. The XCT, cardiopulmonary bypass time and skin-to-skin times of the enrolled cohort of 280 patients in whom implantation of the study valve via minimally invasive surgery was attempted were 52.6 (SD: 18.1), 80.2 (SD: 26.3) and 174.8 (SD: 44.3) min, respectively.

### Haemodynamic results

**Figure 3:**
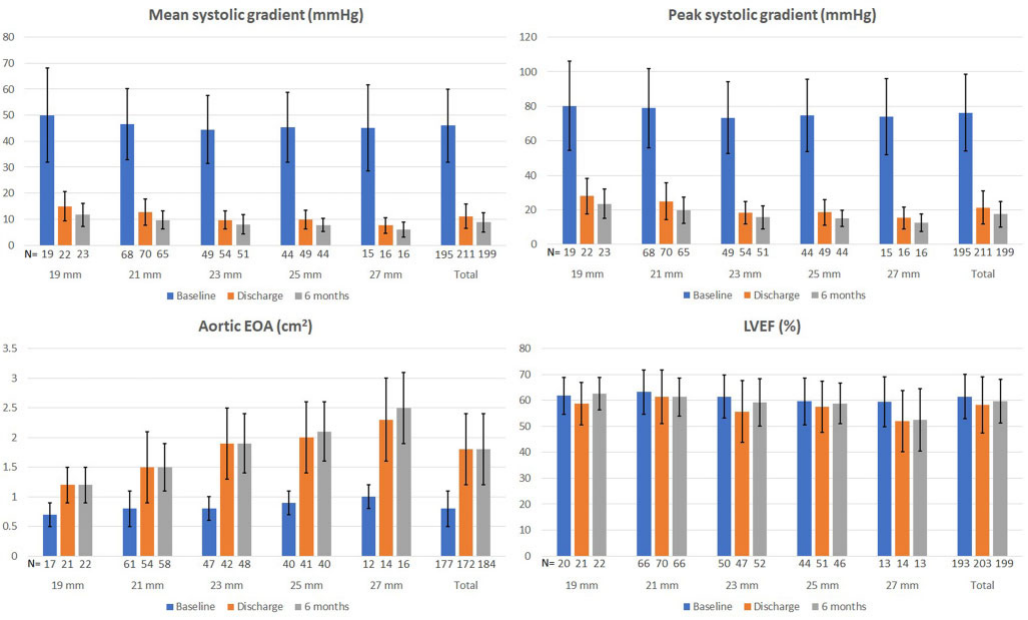
Mean systolic gradient, peak systolic gradient, aortic effective orifice area and left ventricular ejection fraction at preoperative baseline, discharge and after 6 months of follow-up. EOA: effective orifice area (in cm^2^); LVEF: left ventricular ejection fraction.

A total of 212 out of 257 eligible patients underwent echocardiography examination at the 6-month follow-up (see Fig. 3). Mean aortic EOA increased from 0.8 (SD: 0.3) cm^2^ at baseline to 1.8 (SD: 0.6) cm^2^ both at discharge and 6 months across all valve sizes (*P* < 0.0001). Left ventricular ejection fraction was 61.5% (SD: 8.6) at baseline and was not significantly different at 6 months [59.7 (SD: 8.5) %; *P* = 0.0692]. Mean systolic gradient decreased from 46.0 (SD: 14.1) mmHg at baseline to 11.1 (SD: 4.7) mmHg at discharge and 8.8 (SD: 3.7) mmHg at 6 months (*P* < 0.0001). Peak systolic gradient decreased from 76.3 (SD: 22.1) mmHg at baseline to 21.4 (SD: 9.5) mmHg at discharge and 17.5 (SD: 7.4) mmHg at 6 months (*P* < 0.0001). Haemodynamic results are summarized in Table 2. Fourteen of the 139 patients with echocardiography core lab-evaluated EOA index at 6 months follow-up (10.1%) had severe PPM.

**Table 2:** Patient haemodynamics

Parameter	Time point	19 mm	21 mm	23 mm	25 mm	27 mm	Total
		*n*: mean ± SD (min, max)	*n*: mean ± SD (min, max)	*n*: mean ± SD (min, max)	*n*: mean ± SD (min, max)	*n*: mean ± SD (min, max)	*n*: mean ± SD (min, max)
Aortic EOA (cm^2^)	Baseline	17: 0.7 ± 0.2	61: 0.8 ± 0.3	47: 0.8 ± 0.2	40: 0.9 ± 0.2	12: 1.0 ± 0.2	177: 0.8 ± 0.3
	Discharge	21: 1.2 ± 0.3	54: 1.5 ± 0.6	42: 1.9 ± 0.6	41: 2.0 ± 0.6	14: 2.3 ± 0.7	172: 1.8 ± 0.6
	6 months	22: 1.2 ± 0.3	58: 1.5 ± 0.4	48: 1.9 ± 0.5	40: 2.1 ± 0.5	16: 2.5 ± 0.6	184: 1.8 ± 0.6
LVEF (%)	Baseline	20: 61.8 ± 7.1	66: 63.2 ± 8.6	50: 61.5 ± 8.3	44: 59.6 ± 9.0	13: 59.5 ± 9.7	193: 61.5 ± 8.6
	Discharge	21: 58.8 ± 8.2	70: 61.4 ± 10.4	47: 55.7 ± 12.0	51: 57.6 ± 9.9	14: 52.0 ± 11.8	203: 58.2 ± 10.8
	6 months	22: 62.6 ± 6.3	66: 61.3 ± 7.4	52: 59.2 ± 9.1	46: 58.8 ± 7.8	13: 52.5 ± 12.0	199: 59.7 ± 8.5
Mean systolic gradient (mmHg)	Baseline	19: 50.0 ± 18.1	68: 46.6 ± 13.7	49: 44.5 ± 13.0	44: 45.3 ± 13.4	15: 45.1 ± 16.5	195: 46.0 ± 14.1
	Discharge	22: 15.0 ± 5.6	70: 12.8 ± 5.0	54: 9.7 ± 3.4	49: 9.8 ± 3.6	16: 7.6 ± 3.0	211: 11.1 ± 4.7
	6 months	23: 11.7 ± 4.4	65: 9.7 ± 3.4	51: 8.0 ± 3.7	44: 7.8 ± 2.6	16: 6.1 ± 2.9	199: 8.8 ± 3.7
Peak systolic gradient (mmHg)	Baseline	19: 80.3 ± 25.9	68: 78.9 ± 22.8	49: 73.4 ± 20.8	44: 74.8 ± 20.9	15: 74.0 ± 22.0	195: 76.3 ± 22.1
	Discharge	22: 28.0 ± 10.4	70: 25.0 ± 10.7	54: 18.4 ± 6.6	49: 18.6 ± 7.3	16: 15.3 ± 6.2	211: 21.4 ± 9.5
	6 months	23: 23.6 ± 8.4	65: 19.7 ± 7.6	51: 15.7 ± 6.7	44: 15.0 ± 4.7	16: 12.6 ± 4.9	199: 17.5 ± 7.4

All data are presented as *n*: mean ± standard deviation.

EOA: effective orifice area; LVEF: left ventricular ejection fraction.

### Safety results

Safety outcomes are summarized in Table 3. Of the primary cohort of 276 patients successfully implanted with the study valve via minimally invasive surgery, 264 of 273 patients (96.7%) had a follow-up at 30 days and 249 of 257 (96.9%) underwent follow-up at 6 months. There were 10 all-cause deaths, 3 early (1.1%) and 7 (2.7%) late. Of the 7 all-cause death events occurring beyond 30 days, 4 (1.6%) were valve-related.

**Table 3:** Patient safety end-points

	Early events (≤30 days)	Late events (>30 days)	Freedom from event at 6 months		
Adverse event or outcome	*n*, m, *n*/*N*	*n*, m, %/LPY	Patients at risk	Prob. event free	95% CI
Mortality	3, 3 (1.1)	7, 7 (6.1)	191	0.960	(0.936, 0.985)
Valve-related mortality	0, 0 (0.0)	4, 4 (3.5)	191	0.984	(0.968, 1.000)
Reoperation	0, 0 (0.0)	2, 2 (1.7)	191	0.992	(0.982, 1.000)
Study valve explant	0, 0 (0.0)	2, 2 (1.7)	191	0.992	(0.982, 1.000)
Thromboembolism	10, 0 (3.6)	2, 2 (1.7)	184	0.956	(0.931, 0.980)
Stroke	8, 8 (2.9)	2, 2 (1.7)	185	0.963	(0.941, 0.986)
TIA	0, 0 (0.0)	0, 0 (0.0)	191	1.000	(1.000, 1.000)
Non-cerebral TE	2, 2 (0.7)	0, 0 (0.0)	190	0.993	(0.983, 1.000)
Valve thrombosis	0, 0 (0.0)	0, 0 (0.0)	191	1.000	(1.000, 1.000)
Endocarditis	0, 0 (0.0)	0, 0 (0.0)	191	1.000	(1.000, 1.000)
All bleeding	1, 1 (0.4)	3, 3 (2.6)	189	0.985	(0.970, 1.000)
Major bleed	1, 1 (0.4)	3, 3 (2.6)	189	0.985	(0.970, 1.000)
All paravalvular leak (OPC)	2, 2 (0.7)	1, 1 (0.9)	190	0.988	(0.975, 1.000)
Major PVL	1, 1 (0.4)	1, 1 (0.9)	191	0.992	(0.980, 1.000)
Haemolysis	0, 0 (0.0)	0, 0 (0.0)	191	1.000	(1.000, 1.000)
Non-structural valve dysfunction (NSVD) other than PVL	0, 0 (0.0)	1, 1 (0.9)	191	0.996	(0.989, 1.000)
Structural valve deterioration (SVD)	0, 0 (0.0)	1, 1 (0.9)	191	0.996	(0.987, 1.000)
Permanent pacemaker implant	18, 18 (6.7)	2, 2 (1.8)	170	0.929	(0.898, 0.960)
Renal failure	6, 6 (2.2)	0, 0 (0.0)	187	0.978	(0.961, 0.995)
Respiratory failure/dysfunction	3, 3 (1.1)	1, 1 (0.9)	190	0.985	(0.971, 1.000)
Deep sternal wound infection	2, 2 (0.7)	2, 2 (1.7)	188	0.985	(0.970, 1.000)
New or worsening conduction disturbance requiring pacemaker implant	17, 17 (6.3)	0, 0 (0.0)	172	0.936	(0.907, 0.966)

PVL: paravalvular leak.

There was a total of 4 major bleeding events, 1 occurring early (0.4%) and 3 occurring late (1.2%). Two of the 4 major bleeding events were related to anticoagulation; these 4 events were all adjudicated as being of non-cardiac origin. There were 2 major PVL events reported, one early (0.4%) and 1 late (0.4%). The early permanent pacemaker implantation rate was 6.7% (18/269); there were 2 additional patients implanted during the late period, making the freedom from permanent pacemaker implantation rate at 6 months to be 92.9%.

Thromboembolic events were reported in 10 patients early (3.6%) and in 2 patients late. There were no early reoperation events and 2 late reoperation events performed in 2 patients: in one patient, the valve was explanted due to PVL; in the other patient, the valve needed to be explanted in order to provide surgical access to a failing mitral valve.

### New York Heart Association functional outcomes

**Figure 4:**
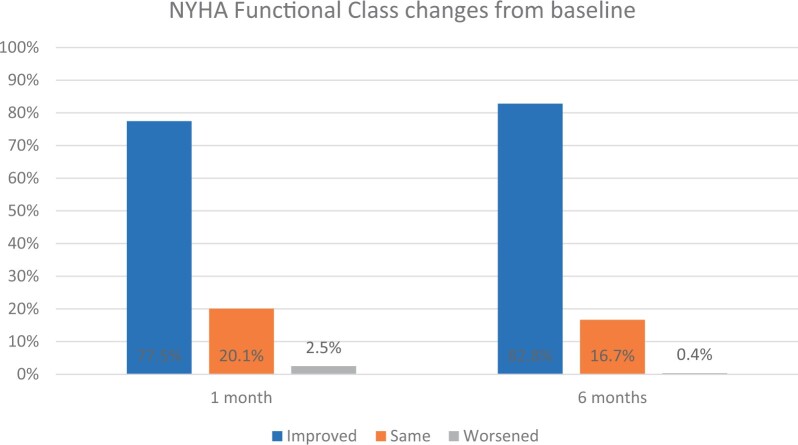
Changes in NYHA functional class from baseline during follow-up. The bars show the proportion of patients whose NYHA functional class improved, stayed the same and worsened at 1 and 6 months of follow-up. NYHA: New York Heart Association.

A summary of NYHA functional classification is presented in Table 4. At baseline, the great majority of patients were either class II (123/275, 44.7%) or class III (135/275, 49.1%). At 6 months, this distribution changed (*P* < 0.0001), with 70.7% (169/239) class I and 26.4% (63/239) class II. At both, the 1 and 6 month follow-ups, a greater proportion of patients improved their NYHA Classification than stayed the same or worsened (*P* < 0.0001 for both time points). At 6 months, 198 of 239 patients (82.8%) improved in NYHA functional class, 40 of 239 patients (16.7%) exhibited no change and 1 of 239 patients (0.4%) worsened compared to baseline (Fig. 4).

**Table 4:** New York Heart Association classification

	Class I	Class II	Class III	Class IV
Baseline	4.0% (11/275)	44.7% (123/275)	49.1% (135/275)	2.2% (6/275)
Six months	70.7% (169/239)	26.4% (63/239)	2.9% (7/239)	0% (0/239)

### Quality of life

Quality of Life improved from baseline to the 6-month follow-up. The quality of life metrics SF-36 Mental Health (*n* = 209), SF-36 Physical Health (*n* = 209) and EQ-5D scores (*n* = 211) increased from 45.5 (SD: 10.4) to 50.1 (SD: 10.1), 42.6 (SD: 8.1) to 47.6 (SD: 8.1) and 0.73 (SD: 0.21) to 0.82 (SD: 0.22), respectively. The distribution of these data within each metric was deemed to be normally distributed, and the mean 6-month scores were statistically significantly better than baseline scores for each variable measured (*P* < 0.0001).

## DISCUSSION

The EDWARDS INTUITY Elite valve revealed a high procedural success rate in this multicentre clinical registry allowing only minimally invasive implantation. Most importantly, procedural time was comparably short and the rate of valve-related adverse events was low. This underlines the previously established role of this rapid-deployment aortic valve in minimally invasive procedures [3, 4, 7]. The application of minimally invasive techniques should be considered as the favourable and primary approach in patients undergoing isolated AVR. High standardization and practice of operative techniques are required to establish a minimally invasive surgical programme for the treatment of aortic valve disease [8, 9]. This may not only reduce procedure-related adverse events but also improve patient satisfaction [10]. Given the currently increasing indication for transcatheter therapies, the proven durability of surgical AVR may be accomplished with reduced operative trauma with a minimally invasive procedure. This is especially true for the INTUITY valve, as this valve is based on the Edwards PERIMOUNT Magna valve, which currently has the longest durability of surgical biologic aortic valves [11]. Recent analysis evaluating valve-in-valve procedures in sutureless and rapid-deployment aortic valves had a very low number of the Intuity valve compared to other valves, also indicating favourable long-term durability [12]. PS and RAT were both performed in this trial, with a slightly numerically increased XCT in the RAT group. A recently published analysis exploring the access-type-related outcome in patients undergoing sutureless and rapid-deployment AVR revealed a very good safety profile for the RAT, and also showed a reduced length of hospital stay in this group [10]. Still, true long-term performance of the Intuity valve is yet to be established.

Postoperative gradients were low and the number of patients suffering from severe PPM was encouraging. This is very important given the decreased survival of patients suffering from severe PPM [13]. We recently demonstrated the INTUITY valve’s promise in patients with a small aortic root [14], and the excellent haemodynamics in the present study in valves of 19 and 21 mm are corroborative. A relevant factor contributing to the excellent flow conditions is the absence of pledgets and a smooth inflow part of the valve due to the subvalvular stent [15]. Furthermore, correct sizing is of paramount importance and only the largest size still fitting should be used to avoid paravalvular regurgitation. This may also contribute to the observed haemodynamic performance. Severe PPM was present in 10.1%, which is numerically lower than a large retrospective analysis for conventional biologic protheses [13]. This is in the line with our recently published single-centre study analysing patients with a small aortic root, which had a substantially lower rate of PPM in the INTUITY group [13, 14].

Despite several benefits of sutureless and rapid-deployment aortic valves, a specific drawback related to the fixation mechanism is the higher risk of permanent pacemaker implantation. The mechanism of injury is comparable to transcatheter AVRs, which may have even a higher risk of pacemaker implantation. The rate of 6.7% in this trial was numerically lower or similar to most earlier experiences and indicates a potential learning curve for the reduction of pacemaker implantations after rapid-deployment aortic valves [16, 17]. The presence of preoperative conduction disturbances was recently identified as a specific risk factor for pacemaker dependency after rapid-deployment aortic valve implantation [18]. Therefore, improved patient selection (e.g. avoidance of patients with a right bundle branch block) should be applied to further decrease the pacemaker rate.

### Limitations

The MISSION Registry was a single-arm study with non-consecutive enrolment, making it susceptible to potential selection bias. As roll-in cases were not allowed, the effect of a learning curve on procedural parameters such as XCT cannot be excluded. Furthermore, performance bias cannot be ruled out despite the emphasis on standardized procedural training across all participating sites.

## CONCLUSIONS

The results of this broad European Registry confirm the EDWARDS INTUITY Elite valve system’s high rate of successful implantation in minimally invasive procedures. The safety profile and haemodynamic performance through the short-term follow-up of 6 months are encouraging.

## PARTICIPATING CENTRES AND LOCAL INVESTIGATORS

Ospedale Del Cuore ‘G.Pasquinucci’: Dr Marco Salinas; Centro Cardiologico Monzino: Prof. Franco Alamanni and Prof. Gianluca Polvani; Santa Maria della Misericordia University Hospital: Prof. Ugolino Livi; Policlinico Universitario ‘Agostino Gemelli’: Prof. Massimo Massetti; Clinica San Gaudenzio: Dr Gian Luca Martinelli; Medizinische Hochschule Hannover: Prof. Dr Malakh Shrestha; Herz- und Gefäss Klinik Bad Neustadt: Prof. Anno Diegeler; Cologne Heart Center: Prof. Dr med. Thorsten Wahlers; Berufsgenossenschäftliche Universitätsklinik Bergmannsheil GmbH, Bochum: Prof. Justus Strauch; Universitätsklinikum Würzburg: Prof. Rainer Leyh; Allgemeines Krankenhaus Wien: Ass Prof. Dr Martin Andreas; Innsbruck Medical University: Ass Prof. Dr Nikolas Bonaros; St. Thomas’ Hospital: Dr Christopher Young; Royal Infirmary of Edinburgh: Dr Renzo Pessoto; Aahrus University Hospital Skejby: Dr Kim Terp; CHU Bocage Central Dijon: Prof. Olivier Bouchot; CHU Haut Levêque Bordeaux: Prof. Louis Labrousse; INCCI Luxembourg: Dr Khaled Chalabi; St. Antonius Hospital: Dr Patrick Klein; Clinico San Carlos: Dr Luis Maroto; Hospital Universitario Virgen de La Arrixaca: Dr Jose Maria Arribas; and Hospitalario Universitario A Coruna: Dr Victor Bautista.

## Notes

Presented at the Annual Techno-College Meeting of the 32nd Annual Meeting of the European Association for Cardio-Thoracic Surgery, Milan, Italy, 18–20 October 2018.

## ABBREVIATIONS

AVR: Aortic valve replacement
EOA: Effective orifice area (in cm^2^)
NYHA: New York Heart Association
PS: Partial sternotomy
PPM: Patient–prosthesis mismatch
PVL: Paravalvular leak
RAT: Right anterior thoracotomy
SD: Standard deviation
XCT: Aortic cross-clamp time
